# Association of a Rare Haplotype in Kinesin Light Chain 1 Gene with Age-Related Cataract in a Han Chinese Population

**DOI:** 10.1371/journal.pone.0064052

**Published:** 2013-06-11

**Authors:** Lu Zhang, Jia-Wei Xu, Xin Qu, Dong-Rui Liu, Ping Liu, Xin-Zhi Zhao

**Affiliations:** 1 Eye Hospital, The First Affiliated Hospital, Harbin Medical University, Harbin, China; 2 Children's Hospital and Institutes of Biomedical Sciences, Fudan University, Shanghai, China; 3 The Third Affiliated Hospital, Harbin Medical University, Harbin, China; University of Illinois at Chicago, United States of America

## Abstract

**Purpose:**

The causal genes for congenital cataract are good candidates for the genetic susceptibility for age-related cataract (ARC). The aim of this study was to investigate association between the polymorphisms in the causal genes for congenital cataract and ARC in a Chinese population. Meanwhile, we performed the replication study for previous identified risk genes for ARC.

**Methods:**

We recruited 212 sporadic Han Chinese patients with age-related cataracts (ARC) and 172 normal controls in this study. We analyzed 31 SNPs from 13 genes which mostly possible contributes the progress of ARC in a Chinese population, comprising 212 cataract patients and 172 controls. Polymorphism-spanning fragments were amplified by using the multiplex polymerase chain reaction (PCR) and genotyped using primer extension method in MassARRAY platform. Allelic and haplotypic difference in the frequencies were estimated using the SHEsis software platform. P-value was adjusted by the Bonferroni correction.

**Results:**

There was no difference in the frequencies of the genotype and allele of the all SNPs between the patients with ARC and the controls. In the haplotypic analysis, the haplotypes consisting of rs7154572, rs7150141 and rs12432994 in Kinesin Light Chain 1 Gene (*KLC1*) showed significant association with ARC (p = 0.000878). A rare haplotype CGT was more frequent in patients (p = 0.000106, and p = 0.00795 after corrected for 75 tests).

**Conclusions:**

Our study provides evidence that the combined effect of three variants within the *KLC1* gene may predispose to ARC, but the precise mechanism needs further investigating.

## Introduction

Cataract is the world's leading cause of blindness, affecting approximately 16 million people [1]. The only effective treatment available is surgery with intraocular lens implantation which is expensive [2]. Cataract is primarily an age-related disorder, age-related cataracts (ARC) usually occur after age 40, and are devided into three main types by their clinical appearance: nuclear, cortical, and posterior subcapsular, which may present alone or in combination [2], [3].

Genetic predisposition and environmental elements contribute to the development of ARC, which are complex and multi-factorial [3]. Age-related cataract is highly heritable. Genetic effects play an important role in nuclear cataracts, explaining almost 50% of the variation in the severity of this disease [4], [5], [6]. The exact genetic mechanism and pathogenesis of age-related cataract remain unclear.

In the past two decades, the identification of the genetic mutations has improved our understanding of the mechanisms of cataractogenesis and normal lens development. The transcription factor Pitx3 is critical for lens development and formation [7], and previous studies have revealed that mutations in *PITX3* are associated with cataracts [8], [9]. MAF is a basic region of leucine zipper transcription factor and R288P substitution within the *MAF* gene affected members of a family with cataract [10]. The common genetic polymorphisms in these genes may also influence the gene function and therefore be involved in the pathophysiology of ARC. For instance, the mutations in *EPHA2* gene would causing the congenital cataract [11], [12], while the common SNPs in this gene were associated with the ARC [13].

Meanwhile, association studies have found candidate genes for susceptibility to ARC. It is reported that the GG genotype of rs8702 in *KLC1* is a high risk for cataract in an Estonian population [14], and a polymorphism of *SOD* is reported associated with an increased risk of cataract in a Chinese population [15]. *EPHA2* was selected as a candidate gene for ARC and three single nucleotide polymorphisms in this gene were studied in an Indian population [16]. The genes contributing to congenital cataract and the coding functional factor in the development of ARC promoted our understanding of the cataract. The common genetic polymorphisms in genes may also influence gene function and therefore be involved in the pathophysiology of ARC. However, more independent replication studies are necessary for the genes.

In order to confirm a possible association between single nucleotide polymorphism (SNP) and the risk of age-related cataract in the Chinese population, we performed a population-based genetic association study including common genetic variations of 31 SNPs in 13 genes, all the selected SNPs were linkage disequilibrium based, and previously reported positive SNPs (Table S1).

## Materials and Methods

### Ethics Statement

The research was approved by the Ethics Committee of the First Affiliated Hospital, Harbin Medical University, Harbin, P.R. China. Informed consent in writing was obtained from each participant personally.

### Subjects

The subjects were 212 sporadic Han Chinese patients with age-related cataracts (ARC) and 172 normal controls (Table 1). All subjects were unrelated individuals from North China and recruited from the Eye Hospital, of the First Affiliated Hospital. Patients with secondary cataracts caused by trauma, uveitis, high myopia, glaucoma, or degenerative ocular diseases were excluded from the study. No patients have any history of hypertension, diabetes, and cancers. Controls were individuals who visited the same hospital for a routine ophthalmic examination and were matched by age-, sex-, and ethnic matched with the ARC patients. The control group included unrelated healthy individuals with no history of cataract, hypertension, diabetes, cancers, or other ocular diseases. All ARC patients and control subjects underwent a full ophthalmic examination, including visual acuity, lens examination with slit lamp biomicroscope after mydriasis, and ophthalmoscopic examination. The main types of ARC clarified among patients (nuclear-65; cortical-55; posterior subcapsular-35; mixed-57). The degrees of cataract in all patients' eyes were CII or CIII according to the Lens Opacities Classification System, version II (LOCS II). All the patients then received phacoemulsification and intraocular lens implantation surgery. The power to detect allele association in population-based samples is 0.70, assuming a frequency of risk allele in population 0.2 and a relative risk of 1.5.

**Table 1:** Demographic data of the patients and controls.

Demographic data	Cataract group	Control group
Male, n (%)	112(52.8)	93(54.1)
Female, n (%)	100(47.2)	79(45.9)
Total	212	172
Age, years (Mean±SD)	68.4±11.4	64.7±7.2

### Blood samples and DNA isolation

We collected 5 ml of venous blood from all the patients and the healthy controls, and stored at −80°C until use. Genomic DNA was extracted from leukocytes using the QIAamp Blood DNA Mini Kit according to the manufacturer's instructions.

### Genotyping

Genomic DNA was diluted to a final concentration of 10–15 ng/uL for the MassARRAY genotyping assays. Polymorphism-spanning fragments were amplified by using the polymerase chain reaction (PCR) and genotyped using the Sequenom MassARRAY SNP genotyping platform (Sequenom, San Diego, CA, USA). The primer sequence and UEP sequence was show in Table S2. PCR and probe sequences are available upon request. Six no-template controls and four duplicated samples in each 384-well format were used for quality control. The genotyping rate for the nine SNPs ranged from 99.1% to 100%. The consistency rate observed in these duplicated samples was 100%.

### Statistical analysis

The case-control study, pairwise linkage disequilibrium (LD) estimation and haplotype reconstruction were performed using a SHEsis software platform (http://analysis.bio-x.cn) [17]. To avoid the problem of multiple testing in genetic analysis of disease association, the permutation test was employed to correct P-values for all the SNPs investigated. The P-values of 1-df test for haplotypic association were corrected using the Bonferroni correction.

## Results

### Single marker association analyses

The genotypic distributions of all 31 markers accorded with Hardy–Weinberg equilibrium (P<0.05). The distributions of allelic frequencies of the tested *SNPs* are shown in Table 2. We did not observe a significance association between single allele and patients, or any difference in the distribution of the age and gender ratio between ARC patients and controls (data not shown).

**Table 2:** Association analysis of 31 makers of 13 candidate genes among patients and controls.

Genes	SNPs	Allele^a^	ARC (%)	Controls (%)	p value	OR	(95% CI)
*KLC1*	rs7154572	**T**	54.4	57.2	0.449	1.12	0.836–1.498
		C	45.6	42.8			
	rs7150141	**A**	10.1	10.5	0.845	0.954	0.591–1.539
		G	89.9	89.5			
	rs12432994	**T**	50	53	0.415	1.13	0.845–1.505
		C	50	47			
*MIP*	rs34287864	**A**	26.7	26.9	0.949	1.011	0.723–1.400
		G	73.3	73.1			
*EPHA2*	rs924201	**T**	72.8	71.3	0.197	1.077	0.775–1.497
		C	27.2	28.7			
	rs3820610	**G**	71	29	0.216	1.231	0.885–1.713
		A	75.1	24.9			
	rs6603856	**A**	15.5	84.5	0.877	0.968	0.642–1.458
		G	15.9	84.1			
	rs3820609	**A**	9.2	11.4	0.314	1.27	0.794–2.041
		C	90.8	88.6			
*ERCC2*	rs1799787	**T**	7	6.5	0.7878	0.082	0.610–1.920
		C	93	93.5			
	rs3916874	**G**	82.4	80.9	0.6216	1.102	0.748–1.623
		C	17.6	19.1			
	rs50871	**G**	28.3	25.3	0.359	1.165	0.840–1.615
		T	71.7	74.7			
*SOD*	rs2070424	**G**	46.8	40.5	0.0988	1.291	0.953–1.748
		A	53.2	59.5			
*PITX3*	rs733283	**A**	22.7	24.8	0.519	1.12	0.795–1.573
		T	77.3	75.2			
*BFSP2*	rs517255	**A**	47.3	55.3	0.035398	1.3803	1.022∼1.864
		C	52.7	44.7			
	rs9877839	**T**	20.2	17.4	0.345489	0.8331	0.570∼1.218
		G	79.8	82.6			
	rs7628262	**C**	32.4	32.9	0.878533	1.0254	0.744∼1.414
		T	67.6	67.1			
*FOXE3*	rs2405922	**A**	41.2	58.8	0.239138	0.8349	0.618∼1.128
		G	45.7	54.3			
*FTL*	rs905238	**G**	41.1	58.9	0.286644	1.183	0.868∼1.612
		A	37.1	62.9			
	rs1039442	**A**	46.1	53.9	0.14744	0.804	0.598∼1.080
		G	51.5	48.5			
*GALK1*	rs8669	**G**	46.3	53.7	0.933917	1.013	0.751∼1.365
		C	46	54			
	rs9367	**C**	45.3	54.7	0.17602	1.225	0.913∼1.643
		T	40.4	59.6			
*GJA3*	rs8000719	**G**	47.3	46.9	0.9133	1.017	0.755–1.370
		C	52.7	53.1			
	rs1886176	**A**	21	20.7	0.921	0.982	0.680–1.416
		C	79	79.3			
	rs17077137	**C**	55.9	52.1	0.2992	1.165	0.873–1.555
		T	44.1	47.9			
	rs7319745	**T**	24.5	25.7	0.699	1.068	0.766–1.489
		C	75.5	74.3			
*GJA8*	rs7541950	**C**	37.9	38.2	0.9374	0.988	0.729–1.338
		T	62.1	61.8			
	rs6674829	**G**	38.5	37.3	0.7282	0.947	0.700–1.285
		A	61.5	62.7			
	rs1532399	**T**	37.8	34.8	0.3971	0.875	0.643–1.191
		G	62.2	65.2			
	rs7544630	**C**	27.1	29.6	0.503	0.887	0.623–1.261
		T	72.9	70.4			
*MAF*	rs8045611	**C**	60.6	54.1	0.108	1.3	0.943–1.794
		T	39.4	45.9			

**a**: means the upper are minor alleles.

### Haplotype association analyses

We calculated linkage disequilibrium (LD) for all pairs of markers in each gene. The LD test for all pairs of markers in *KLC1* showed strong LD (r^2^>0.8) for rs7154572, rs7150141 and rs12432994 (Figure S1). Our results indicated that these three SNPs were in one LD block with a haplotype frequency. The overall haplotype frequency revealed significant differences between cases and controls (p = 0.000878). The C-G-T haplotype which was more frequent in patients, proved to be significantly associated with ARC (p = 0.000106) (Table 3). The association remained significant even after a stringent Bonferroni correction (p = 0.00795, corrected for 75 tests). However, the haplotypes in the remaining genes showed no significance association with ARC (data not shown).

**Table 3:** *KLC1* haplotype frequencies and p values among ARCs and controls.

*KLC1* haplotype	Case(freq)	Control(freq)	Chi2	p value	Odds Ratio	95%CI
C-A-C	35.74(0.093)	32.08(0.101)	0.115	0.73438	0.917	0.555–1.515
C-G-C	122.55(0.319)	103.92(0.327)	0.04	0.842135	0.968	0.704–1.331
T-G-C	28.48(0.074)	14.00(0.044)	2.802	0.094225	1.744	0.903–3.367
T-G-T	175.30(0.457)	165.08(0.519)	2.675	0.102015	0.779	0.578–1.051
C-G-T	17.67(0.046)	0.00(0.000)	15.044	0.000106	–	–

## Discussion

In this study, we tested the association between ARC and 31 SNPs located in 13 genes in a Han Chinese population. We found no significant single marker associated with ARC patients. We found that the haplotype C-G-T of *KLC1* is highly associated with ARC, although the frequency is low in both cases and controls. Moreover the three SNPs genotyped showed high linkage disequilibrium. Our results suggest that the interaction of the potential functional variants among genetic variants within the *KLC1* gene may contribute to an increased risk of ARC, and this supports the inference that *KLC1* is a susceptibility gene for age-related cataract [14].

*KLC1*, a member of the kinesin superfamily, is an important ATP-dependent intracellular transport protein [18]. Like Parkinson's disease and Alzheimer's disease, ARC is a conformational disease directly or indirectly linked to impaired kinesin-mediated transport [19]. Moreover, kinesin has been co-localized with amyloid-β precursor protein(APP) and APP-like proteins in the lens, and the similarity of the cargo-trafficking in neurons and lens fiber cells suggests that impaired transport could be a potential overlapping pathogenic mechanism for ARC in these tissues [20]. Kinesin is composed of two heavy chains which can form tetramers with two light chains, the heavy chains contain the microtubule-binding and ATP-utilizing domains that bind to and are responsible for movement along the microtubules [21]. Hence, kinesin light chains bind to the heavy chains and contain the cargo-binding domain of the complex, kinesin may play an important role in the development of ARC.

In our study, C-G-T haplotype in *KLC1* was observed 4.6% of ARC, whereas it was not observed it in the control group. A previous study revealed that rare variants, as well as common variants, can also contribute to the multifactorial inheritance of common diseases [22]. *KLC1* was reported to be a novel susceptibility gene for age-related cataract, and the GG genotype of rs8702 was also significantly associated with cataract patients [14]. Although each single variation may not be sufficient to confer susceptibility to ARC in our population, the combination of a few variants within a gene could have an additive effect on the risk of disease.

In conclusion, our study provides a suggestion that the combined effect of three variants within the *KLC1* gene may predispose to ARC, but the precise mechanism needs further investigating.

## Supporting Information

Figure S1 **LD plot for** ***KLC1.*** The LD plot was generated by SHEsis. r^2^ between maker pairs is indicated by the shaded matrices. This figure shows that strong LD was observed between rs7154572, rs7150141 and rs12432994.
(TIF)

Table S1 **Selected genes and SNPs detail.**
(DOC)

Table S2 **Primer sequences and UEP primers used for MassARRAY IPLEX Genotyping.**
(DOC)
