# Supplementary material for: Association of a Rare Haplotype in Kinesin Light Chain 1 Gene with Age-Related Cataract in a Han Chinese Population
Source: PLoS One. 2013 Jun 11;8(6):e64052. doi: 10.1371/journal.pone.0064052 (PMC3679130; doi:10.1371/journal.pone.0064052)
Supplement: Table S1 — Selected genes and SNPs detail. (DOC) [file pone.0064052.s002.doc]

**Table S1: Selected genes and SNPs detail**

| **Genes** | **SNPs** | **Allele ^a^** | **Chromosome Locations** | **Gene Functional categories** |
| --- | --- | --- | --- | --- |
| *KLC1* | rs7154572 | **T** | Chr14:104105414 | microtubule motor activity, motor activity, protein binding |
|  |  | C |  |  |
|  | rs7150141 | **A** | Chr14:104129352 |  |
|  |  | G |  |  |
|  | rs12432994 | **T** | Chr14:104138639 |  |
|  |  | C |  |  |
| *MIP* | rs34287864 | **A** | Chr12:55144584 | structural constituent of eye lens, transporter activity, water channel activity |
|  |  | G |  |  |
| *EPHA2* | rs924201 | **T** | Chr1:16318936 | ATP binding, ephrin receptor activity, protein binding, transmembrane receptor protein tyrosine kinase activity |
|  |  | C |  |  |
|  | rs3820610 | **G** | Chr1:16452998 |  |
|  |  | A |  |  |
|  | rs6603856 | **A** | Chr1:16460840 |  |
|  |  | G |  |  |
|  | rs3820609 | **A** | Chr1:16467645 |  |
|  |  | C |  |  |
| *ERCC2* | rs1799787 | **T** | Chr19:45856144 | Aging, induction of apoptosis, apoptotic process, gene expression, DNA repair |
|  |  | C |  |  |
|  | rs3916874 | **G** | Chr19:45856926 |  |
|  |  | C |  |  |
|  | rs50871 | **G** | Chr19:45862515 |  |
|  |  | T |  |  |
| *SOD* | rs2070424 | **G** | Chr21:33039320 | antioxidant activity, copper ion binding, superoxide dismutase activity |
|  |  | A |  |  |
| *PITX3* | rs733283 | **A** | Chr10:103996576 | DNA binding, protein binding |
|  |  | T |  |  |
| *BFSP2* | rs517255 | **A** | Chr3:133118054 | structural constituent of eye lens, structural constituent of cytoskeleton |
|  |  | C |  |  |
|  | rs9877839 | **T** | Chr3:133148223 |  |
|  |  | G |  |  |
|  | rs7628262 | **C** | Chr3:133189192 |  |
|  |  | T |  |  |
| *FOXE3* | rs2405922 | **A** | Chr1:47646271 | DNA binding, bending, transcription factor binding |
|  |  | G |  |  |
| *FTL* | rs905238 | **G** | Chr19:49465384 | ferric iron binding, identical protein binding, oxidoreductase activity |
|  |  | A |  |  |
|  | rs1039442 | **A** | Chr19:49471330 |  |
|  |  | G |  |  |
| *GALK1* | rs8669 | **G** | Chr17:73750859 | ATP binding, galactokinase activity, galactose binding |
|  |  | C |  |  |
|  | rs9367 | **C** | Chr17:73753661 |  |
|  |  | T |  |  |
| *GJA3* | rs8000719 | **C** | Chr13:19607214 | gap junction channel activity |
|  |  | G |  |  |
|  | rs1886176 | **A** | Chr13:19613801 |  |
|  |  | C |  |  |
|  | rs17077137 | **C** | Chr13:19619985 |  |
|  |  | T |  |  |
|  | rs7319745 | **T** | Chr13:19623636 |  |
|  |  | C |  |  |
| *GJA8* | rs7541950 | **C** | Chr1:145842605 | channel activity |
|  |  | T |  |  |
|  | rs6674829 | **G** | Chr1:145845288 |  |
|  |  | A |  |  |
|  | rs1532399 | **T** | Chr1:145848068 |  |
|  |  | G |  |  |
|  | rs7544630 | **C** | Chr1:145849174 |  |
|  |  | T |  |  |
| *MAF* | rs8045611 | **C** | Chr1:145849174 | sequence-specific DNA binding transcription factor activity |
|  |  | T |  |  |

**^a^** means the upper are minor alleles
