# Supplementary material for: Association of a Rare Haplotype in Kinesin Light Chain 1 Gene with Age-Related Cataract in a Han Chinese Population
Source: PLoS One. 2013 Jun 11;8(6):e64052. doi: 10.1371/journal.pone.0064052 (PMC3679130; doi:10.1371/journal.pone.0064052)
Supplement: Table S2 — Primer sequences and UEP primers used for MassARRAY IPLEX Genotyping. (DOC) [file pone.0064052.s003.doc]

**Table S2. Primer sequences and UEP primers used for MassARRAY IPLEX Genotyping**

| SNP ID | Forward primer | Reverse primer | UEP Primer |
| --- | --- | --- | --- |
| rs8000719 | ACGTTGGATGAGGAAAATGGTGCATGCCTA | ACGTTGGATGGGAGGAAGCCATTTTCTGTC | GCATGCCTACGCATA |
| rs9877839 | ACGTTGGATGAAGTGTCAGCAGGTTGGGAG | ACGTTGGATGATAGGATTCCACCAGCAGAC | GGTTGGGAGGCCCAG |
| rs2405922 | ACGTTGGATGTTCCAACTGGCTTGTGAGAC | ACGTTGGATGGCCAATTTCAAACTACGACC | CTCCCAACACTGCTTTT |
| rs8669 | ACGTTGGATGACTCACCGTGGGAGGAGAC | ACGTTGGATGCGCTCAGAACACTCACACTC | GGGGTGGAGTAGTCCCT |
| rs905238 | ACGTTGGATGTTACCCACACGCCTGCTTG | ACGTTGGATGTGGTACATCTGTCGCAGCC | TCCGCTGCCTCTGTCCCG |
| rs1532399 | ACGTTGGATGGAGGACAGGAGACAGAAGAT | ACGTTGGATGCAAGCTTACGTAGGGCAAGA | AAGATCATGTTGGCACCT |
| rs924201 | ACGTTGGATGCACAGCACTCACCACATTCC | ACGTTGGATGACAAGGACTGGGCTTTTGAG | ACCCATATCCAGCTCCACAG |
| rs1039442 | ACGTTGGATGTCAGACATGACACTTGGTGG | ACGTTGGATGTAAGAGGATGAGCTCAGACC | TGCGGTGACGTCCCCCCAGT |
| rs3758549 | ACGTTGGATGCTCCTAGGAGAAAACCCATC | ACGTTGGATGACCTGTATTACCTGGAAGTG | CTCTTCTTGTCCTCTTCTCAC |
| rs2070424 | ACGTTGGATGCTCAGTTACTGAGTTATGCC | ACGTTGGATGTATATCAGAGGCCTTGGGAC | TGAGTTATGCCTGTTAATTAC |
| rs1886176 | ACGTTGGATGTAAAGGGACTGCAGGATACC | ACGTTGGATGAGAAATCTGGTCGCTGAGGG | CCCCCCTGTTGCTTTACCACTA |
| rs6674829 | ACGTTGGATGCAGCCATATGTTACAGACCC | ACGTTGGATGAGGAAGGTCTTCTAGGACAC | GACCCTGAAACATTTTCATTCC |
| rs7628262 | ACGTTGGATGCAGGAGGAGACTTTTTACAC | ACGTTGGATGTCAAGATGGTGTCGTGACAG | CCCCGTTACACCCTTATGACCACT |
| rs7541950 | ACGTTGGATGAGTGTGTTCAATTTAAAGG | ACGTTGGATGAAAGGGGATTTTAACACCAC | CCTCATTCTGAAGTTATGTCCTCTC |
| rs6603856 | ACGTTGGATGTCGCTACACACTCTAACTCG | ACGTTGGATGACAGGATGCTAGTCGAGTCT | GGCCATCGTATATCCTCGCACCATCC |
| rs3916874 | ACGTTGGATGCAGGCAGAGCCAATCAGAG | ACGTTGGATGTCCTTGCATTGGCTCCACAC | CCAATGCCCTGAGACTTCCCATATGACA |
| rs7544630 | ACGTTGGATGCACTACTTTGGAGTGTTTTG | ACGTTGGATGTGAGCCAAAAGAATGGTCAG | TTTTTTTAAATCATCAGTCCAGGAGATA |
| rs517255 | ACGTTGGATGCTCTGCTTCTCCATCTGTTG | ACGTTGGATGGGAGAGGCTTACCAGAAAAC | GGGTAAAACTGAGAAATAAAAACAACAA |
| rs34287864 | ACGTTGGATGCCTTTCCTCAAGCGATCTAC | ACGTTGGATGAGACAATGGGCCACTGCACT | CCTCCCGCCCGGCCT |
| rs1799787 | ACGTTGGATGGGTTACAAGTGTGGCTGGTG | ACGTTGGATGCCCAACTCAGACACAGCATC | TGGGACAGCCTCACGC |
| rs8045611 | ACGTTGGATGGGTTTAGATCACCAACTTCC | ACGTTGGATGAAGAAAATGCCTCTGAGCCC | TCCCTGCCATTTCCTGA |
| rs50871 | ACGTTGGATGATGAGGAAGGATGATCCAGG | ACGTTGGATGACCTTTGTCATTCTCTACCC | GATGATCCAGGTGAAAGA |
| rs3820609 | ACGTTGGATGAGAGGAGATAAGGCCTGACC | ACGTTGGATGACAACACATAGACCCACAGC | CACTTCAAAAGCCAGAAAC |
| rs7319745 | ACGTTGGATGAAACACAGGGTCTCCTGTCC | ACGTTGGATGGGAGTCTGACACATGGAACT | TGTCGGGAAGTCACACAACA |
| rs3820610 | ACGTTGGATGAATGTCTCGGCCTTCCTGCT | ACGTTGGATGAGGGCAGACCCAGGTGAGTT | GTGTGAGCACCAGCCTCTGCG |
| rs12432994 | ACGTTGGATGTGTGTAAAGGCACAAAGCGG | ACGTTGGATGGAATGAACACTAACCTCCTC | GGGGACAAAGCGGAGAGAACT |
| rs9367 | ACGTTGGATGTCTTCCAAACTTGACCGCAC | ACGTTGGATGGAGGCTCCGGGAGAGGAGT | GCGAACCCTGCCCCACCCCCGCCA |
| rs7154572 | ACGTTGGATGGGGAATTATAATGATAGGTC | ACGTTGGATGGGAGCATTTTCAAATGTGTAA | TTATAATGATAGGTCATCAAACTA |
| rs17077137 | ACGTTGGATGCATGTCTGTAGTAAGCACAC | ACGTTGGATGATCCCACAGTCATTATCCTC | CCCATATACAATACACTTTGCACGC |
| rs7150141 | ACGTTGGATGGTTTTTAACCATCTTGCCCC | ACGTTGGATGAAGAGAAGTGGATATGGTTG | CCCCTTAAAATATATTTTTATGTATCT |
| rs733283 | ACGTTGGATGTCTCATCTCTCCATTTAGCC | ACGTTGGATGTGGAGGCATAACCTGGAACT | TCTATCATCTCTCCATTTAGCCCCTGAA |
